# An overview of the characteristics of the novel avian influenza A H7N9 virus in humans

**DOI:** 10.3389/fmicb.2015.00140

**Published:** 2015-03-05

**Authors:** Kei-Xian Tan, Sabrina A. Jacob, Kok-Gan Chan, Learn-Han Lee

**Affiliations:** ^1^Jeffrey Cheah School of Medicine and Health Sciences, Monash University MalaysiaBandar Sunway, Malaysia; ^2^School of Pharmacy, Monash University MalaysiaBandar Sunway, Malaysia; ^3^Division of Genetics and Molecular Biology, Institute of Biological Sciences, Faculty of Science, University of MalayaKuala Lumpur, Malaysia

**Keywords:** characteristics, novel, avian influenza A, H7N9, virus

## Abstract

The novel avian influenza A H7N9 virus which caused the first human infection in Shanghai, China; was reported on the 31st of March 2013 before spreading rapidly to other Chinese provinces and municipal cities. This is the first time the low pathogenic avian influenza A virus has caused human infections and deaths; with cases of severe respiratory disease with pneumonia being reported. There were 440 confirmed cases with 122 fatalities by 16 May 2014; with a fatality risk of ∼28%. The median age of patients was 61 years with a male-to-female ratio of 2.4:1. The main source of infection was identified as exposure to poultry and there is so far no definitive evidence of sustained person-to-person transmission. The neuraminidase inhibitors, namely oseltamivir, zanamivir, and peramivir; have shown good efficacy in the management of the novel H7N9 virus. Treatment is recommended for all hospitalized patients, and for confirmed and probable outpatient cases; and should ideally be initiated within 48 h of the onset of illness for the best outcome. Phylogenetic analysis found that the novel H7N9 virus is avian in origin and evolved from multiple reassortments of at least four origins. Indeed the novel H7N9 virus acquired human adaptation via mutations in its eight RNA gene segments. Enhanced surveillance and effective global control are essential to prevent pandemic outbreaks of the novel H7N9 virus.

## INTRODUCTION

The influenza A virus is a member of the Orthomyxoviridae family that consists of an enveloped negative-sense, single-stranded RNA; while its whole genome is made up of eight RNA gene segments. The first, second and third RNA segments are transcriptase-associated proteins such as polymerase basic 1 (PB1), polymerase basic 2 (PB2), and polymerase A (PA). The fourth, fifth, sixth, seventh, and eighth segments are hemagglutinin (HA), nucleocapsid protein (NP), neuraminidase (NA), and membrane or matrix protein (M1 and M2), followed by non-structural proteins (NS1 and NS2; Lamb and Choppin, 1983). Influenza A viruses can evolve via reassortment, which is the exchanging and mixing of eight RNA gene segments from two different influenza strains in a single host to form a new influenza virus (Liu et al., 2013a). Furthermore, Influenza A viruses are categorized into different subtypes based on the combination of two surface proteins, which is hemagglutinin and NA. There are currently H1 to H18 and N2 to N10 surface proteins identified (Nagy et al., 2012; Chen et al., 2013; Shi et al., 2013a; To et al., 2013).

The novel avian-origin influenza A virus that is known as the H7N9 virus and caused severe and fatal respiratory disease was identified in Shanghai, China (Li et al., 2014). Although many avian influenza virus subtypes such as H5N1, H7N1, H7N7, H7N2, H7N3, and H9N2 were found to be capable of infecting humans; the low pathogenic avian influenza A H7N9 virus had never caused human infections with a fatal outcome until the outbreak in Qi et al. (2013); Richard et al. (2013), and Shi et al. (2013a). This novel H7N9 virus is the first H7N9 subtype to cause human infections, and by May 16 2014 had caused a total of 440 human infections including 122 deaths (Watanabe et al., 2014). In contrast, this novel virus is lowly pathogenic in poultry (Richard et al., 2013); which is the main source of infection (Li et al., 2014). This literature review will discuss the epidemiology, origin and diversity, virology, clinical characteristics, treatment, control, and prevention of the novel H7N9 virus.

## EPIDEMIOLOGICAL CHARACTERISTICS

**FIGURE 1:**
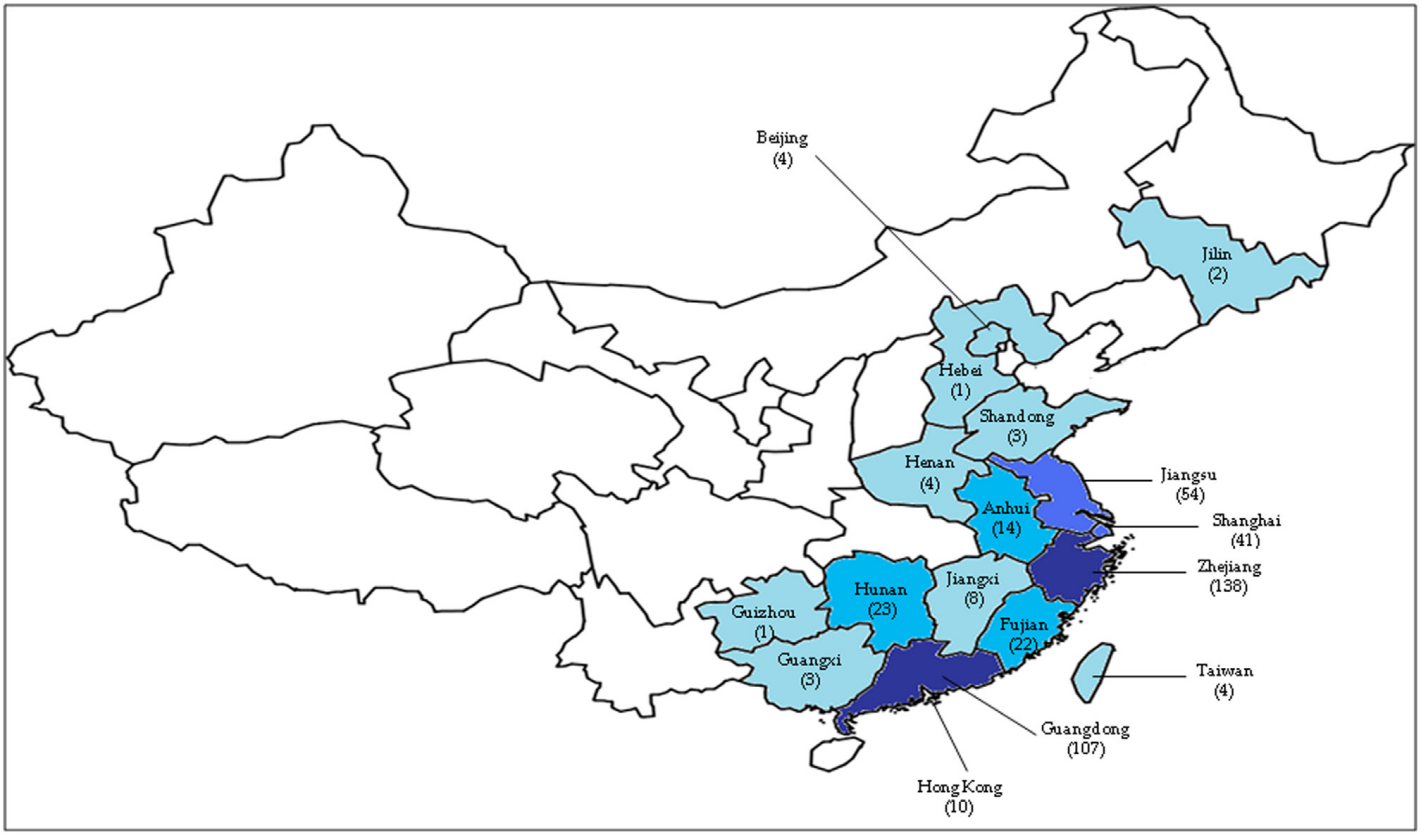
**The geographic areas of confirmed cases of human infection with avian influenza A H7N9 virus, as of 16 May 2014 (total of 440 cases).** The number of cases in each province is based on data reported by the Centre for Health Protection, Hong Kong PRC SAR (http://www.chp.gov.hk/en/index.html). The darker the blue color, the higher the number of cases. One case detected in Malaysia was not shown in this figure.

The first human case was reported in Shanghai on the 31st of March 2013 (CDC, 2014). Shanghai was affected the most by this novel virus due to its location along the Asian-Australian flyway, where migratory birds transit at the wetlands. This city also has the highest densities of both people and poultry (Bingsheng and Yijun, 2007). After the emergence of the infection in Shanghai, subsequent cases were detected in neighboring cities such as Jiangsu, Zhejiang, and Anhui around the lower Yangtze River delta in the following months (**Figure 1**; Liu et al., 2013a). This novel virus then spread to other regions of China such as Beijing, Henan, Hunan, Jiangxi, Fujian, and Shandong (Butler, 2013). However, among all the provinces, most cases were reported in Shanghai, Zhejiang and Jiangsu; provinces located in the eastern part of China (Peng et al., 2013). There was no H7N9 infection cases found in other countries outside China except for one case that was reported in Taipei on the 24th of April, whereby the patient had recently returned from Jiangsu (Chang et al., 2013). Previously, H7N9 viruses were found in chickens in the USA (Pasick et al., 2012) and birds in South Korea (Kim et al., 2012). However, it had never caused human infections until the outbreak in 2013 (Qi et al., 2013; Richard et al., 2013; Shi et al., 2013a; Li et al., 2014). Kageyama et al. (2013) reported that H7 subtypes have never caused fatal cases except the H7N7 subtype, which caused one fatality in the Netherlands in 2003; while human infections with the N9 subtype had never been reported in any country (Gao et al., 2013a; Liu et al., 2013a; Shi et al., 2013a).

Toward the end of April 2013, the human cases of H7N9 infections increased significantly, reaching 125 confirmed cases. Researchers detected that the main source of the H7N9 virus infection was through exposure to infected live poultry markets or contaminated surroundings (Chen et al., 2013; Peng et al., 2013; Richard et al., 2013; Shi et al., 2013b; van Riel et al., 2013). This prompted the Chinese government to suspend live poultry markets in many provinces such as Shanghai and Zhejiang (Murhekar et al., 2013; Xu et al., 2013a); and halt the trading of live birds carrying the virus. These measures effectively prevented further spread of the virus as a rapid decline in new human H7N9 infections was observed during the following 2 weeks (Chowell et al., 2013; Yu et al., 2013). The number of confirmed human H7N9 cases increased significantly, however, in January and February 2014, with more than 30 new cases over several consecutive weeks (WHO, 2013). This could possibly be as a result of the fall in temperature in the winter of 2013, and/or the reopening of the live poultry markets (Watanabe et al., 2014). By the 16th of May 2014, a total of 440 human H7N9 cases were confirmed with 122 associated deaths (unofficial statement ^1^). Of these, 425 cases occurred in China ^2^, while the remaining 15 were exported cases ^3^ (**Figure 1**).

The age of infected patients ranged from 2 to 91 years old while most of them were older instead of young adults with a median age of 61 years. Recent studies reported that patients were mostly older adults due to several factors. Firstly, children with H7N9 presented mostly with mild or asymptomatic infections that might have been under diagnosed (Mei et al., 2013; Xu et al., 2013b). Secondly, most of the adult patients were poultry workers or visitors who had prolonged exposure to the H7N9 virus in a larger dose. Moreover, researchers found that older adults have a higher risk of comorbid illnesses and a weaker immune response toward the H7N9 virus due to the immunological phenomenon of ‘original antigenic sin’ (Kim et al., 2009; Skowronski et al., 2013). However, population immunity toward this novel H7N9 virus is low as it only emerged recently. Hence, individuals of any age are susceptible to it (Shi et al., 2013b; Li et al., 2014).

Most of the infected individuals were males, with a male-to-female ratio in urban and rural areas of 2.4:1 and 1.6:1 respectively. This was mostly due to gender-based differences in exposure to poultry as opposed to differences in immunity (WHO, 2013). Additionally, older men were found to be weaker than older women due to their shorter mean life-span (Zhuang et al., 2013). However, Liu et al. (2013b) found that susceptibility toward the novel H7N9 virus was not affected by gender, race, occupation, income level, and educational background.

It was noted that more than two- thirds of infected patients had occupational or recent exposure to poultry in the 7 days before the onset of illness (Shi et al., 2013a; van Riel et al., 2013). This was further supported when gene sequences which were highly similar to the H7N9 virus were found in ducks, chickens, pigeons and environmental samples from live poultry markets in affected regions in China. More studies should be done to investigate the possibility of human H7N9 infections caused by the consumption of inadequately cooked poultry products and contact with wild birds, as documentation of such cases is insufficient.

To date there is no definitive evidence on sustained person-to-person transmissions, although two family clusters were reported. This was because there were no other cases reported among healthcare workers or those in close contact with infected patients (Ramos et al., 2013; van Riel et al., 2013; Yu et al., 2013). Moreover, H7N9 human cases were discovered in different areas of China and were not epidemiologically related (Gao et al., 2013a; Richard et al., 2013). Some researchers also reported that most of the H7N9 patients with severe or fatal cases were obese, smokers, chronic drug users, had comorbidities, lung or immunosuppressive diseases; delayed antivirus treatments, and increasing age (Gao et al., 2013a; Peng et al., 2013; Shi et al., 2013a). Studies have also found that infected patients who have a higher risk of complications such as acute respiratory distress syndrome (ARDS) and respiratory failure, were those who were less than 5 years or more than 64 years of age; or those who had underlying medical illnesses (Li et al., 2014). It has also been postulated that this novel H7N9 virus might have a seasonal pattern, given that the number of human H7N9 cases dwindled over the summer after its peak in spring, between March 28 and April 18 in Liu et al. (2013c). An increase, however, was noted in early October 2013, at which more new H7N9 infections were reported, coinciding with the arrival of winter in China (CDC, 2014).

One of the reasons for the emergence of the novel H7N9 virus that caused a human epidemic in China is the high population of human and poultry (van Riel et al., 2013). Given that the novel H7N9 virus originated from live poultry markets that gather many different types of wild birds and poultry species, reassortment could occur easily among the different subtypes of avian influenza viruses to produce the novel H7N9 virus (Chang et al., 2013; Chen et al., 2013; Gao et al., 2013b; Liu et al., 2013a; Xiong et al., 2013a). Furthermore, the selective pressure induced by the widespread use of the H5N1 vaccine could cause the emergence of the H7N9 virus, as H5 and H7 are the common subtypes that generally cause poultry outbreaks. China uses more than 90% of the H5N1 vaccine worldwide (Swayne, 2012). While the H5 vaccine efficiently restrains the circulating clade of H5 virus in China, this vaccine is unable to prevent the emergence of antigenically drifted H5 and non-H5 viruses. Furthermore, poor biosecurity measures at farms and wet markets permit the easy intrusion and strengthening of a new virus reassortant. The mechanism of spread of the H7N9 outbreak in 2013 between different geographical regions, however, remains unclear; and as such speculation over the roles of migratory birds, poultry and human beings warrant further investigation.

## ORIGIN AND DIVERSITY

**FIGURE 2:**
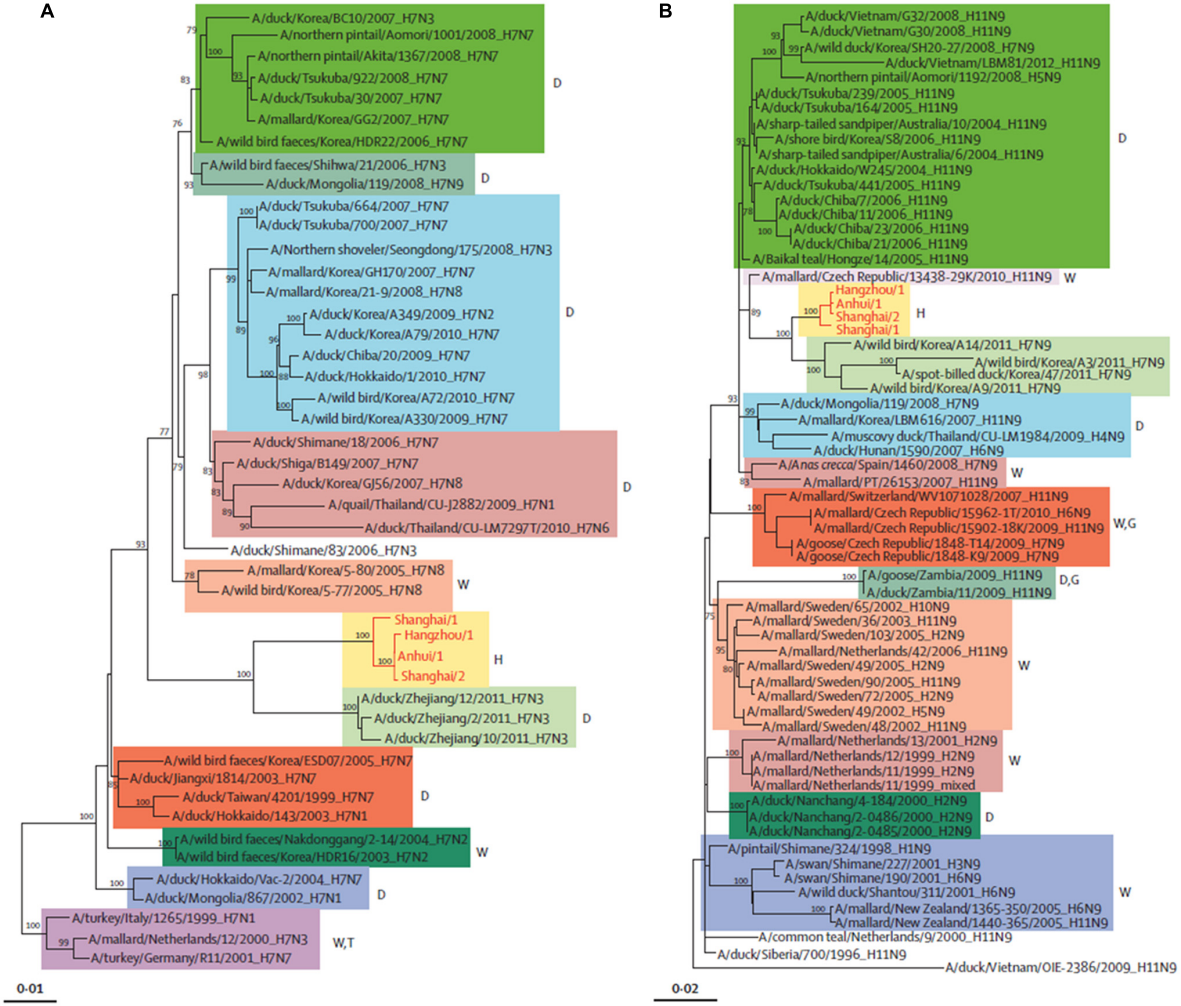
**Phylogenetic trees of H7 **(A)** and N9 **(B**).** The H7N9 viruses are shown by red lines and text. D, duck; W, wild bird; H, human being; T, turkey; G, goose. Adapted from Liu et al. (2013a).

**FIGURE 3:**
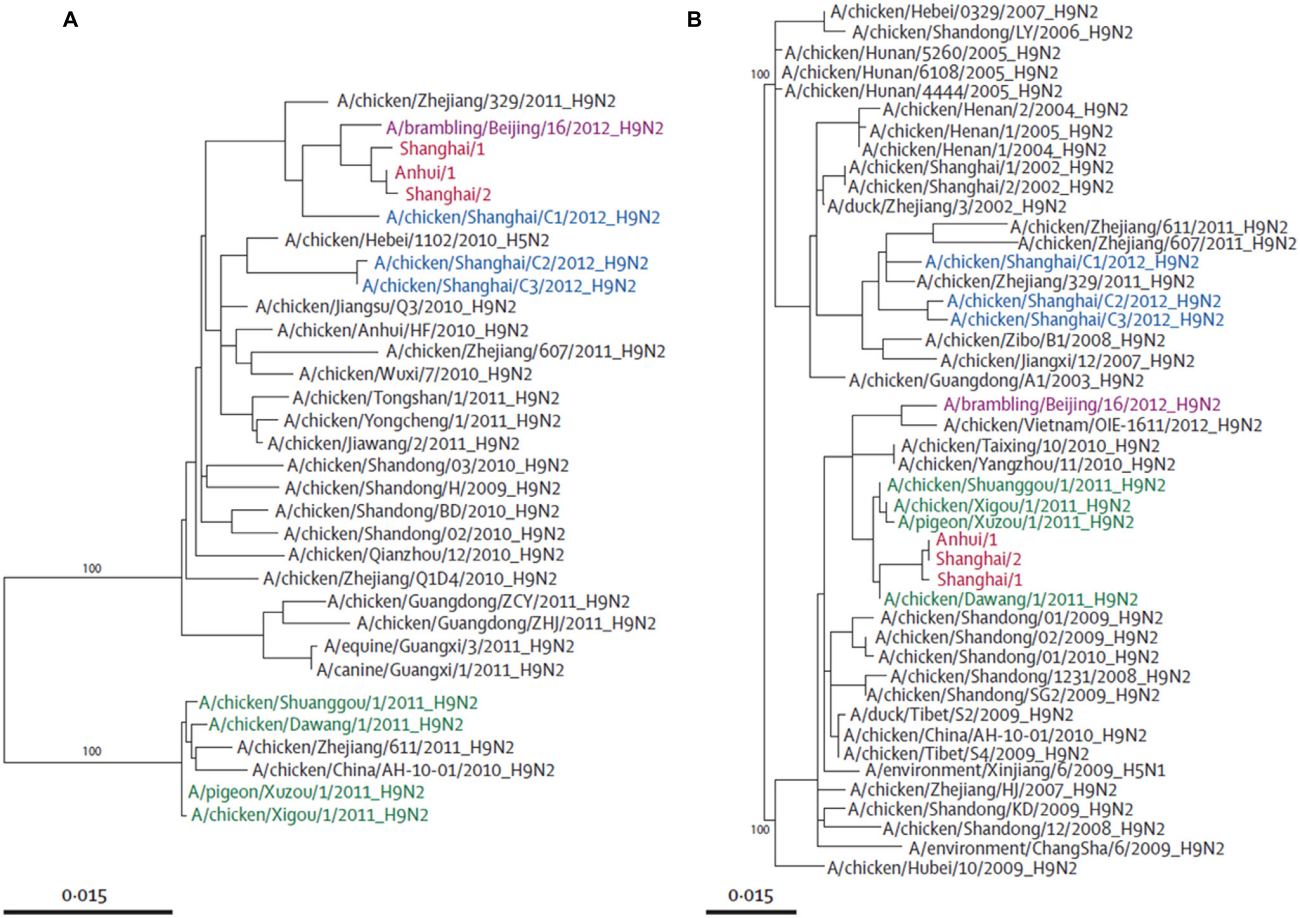
**Phylogenetic trees of PB2 **(A)** and NS **(B)**.** Avian influenza A H7N9 viruses are shown in red, brambling viruses in pink, viruses isolated from Shanghai in 2012 in blue, and viruses isolated from Jiangsu province in green. Adapted from Liu et al. (2013a).

Phylogenetic studies have shown that the novel H7N9 virus is avian in origin and is a result of multiple reassortments of avian influenza viruses from at least four origins (**Figures 2** and**3**; Gao et al., 2013b; Liu et al., 2013a). The gene encoding for HA of the H7 subtype was most closely related to the avian influenza A H7N3 viruses from domestic ducks in Zhejiang (**Figure 2A**); while the NA gene segment of the N9 subtype was most closely related to H7N9 viruses from wild birds in South Korea (**Figure 2B**); and six internal genes originated from two different groups of avian influenza A H9N2 viruses in brambling birds or chickens in Shanghai, Zhejiang or Beijing, China (**Figure 3**; Kageyama et al., 2013; Knepper et al., 2013; Liu et al., 2013a; Peng et al., 2013; Qi et al., 2013; Richard et al., 2013; Shi et al., 2013a,b; Tharakaraman et al., 2013; Watanabe et al., 2013; WHO, 2013; Li et al., 2014). Many researchers indicated that the internal genes of this novel virus which are PA, PB1, and PB2; originated from the H9N2 virus from bramblings in Beijing (**Figure 3A**); while M, NP and NS genes originated from the H9N2 virus from chickens in eastern China (**Figure 3B**; Chang et al., 2013; Chen et al., 2013; Gao et al., 2013b; Liu et al., 2013a; Xiong et al., 2013a). Furthermore, all studies found that gene reassortment happened in an avian host since all 8 gene segments of novel H7N9 viruses are avian in origin (; Chang et al., 2013; Chen et al., 2013; Gao et al., 2013b; Liu et al., 2013a; To et al., 2013; Xiong et al., 2013a). Studies have also reported that all the isolated novel H7N9 strains share a common ancestor as they consist of highly similar nucleotide and amino acid sequences based on genetic analysis (Kageyama et al., 2013).

The novel H7N9 virus originated from new virus reassortants, at which each of the hemagglutinin, NA and internal genes from H7N3, H7N9, and H9N2 viruses respectively (**Figures 2** and **3**) circulated silently in domestic poultry for a period of time, and then reasserted silently to form this novel H7N9 virus; which gained the ability to infect human beings in the Yangtze River Delta (Peng et al., 2013; WHO, 2013).

Mammalian adaptation of the novel H7N9 virus might be acquired via mutations and genetic changes that occurred in domestic poultry to cause poultry- to- human transmissions (Shi et al., 2013a). Apart from that, novel H7N9 strains isolated from the first patient (A/Shanghai/1/2013) were found to be phylogenetically different from other human and avian isolates found later. This proved that this new reassortant obtained this degree of diversity after circulating for a period of time, and also showed that there were two or more introductions into humans. It is thus postulated that further adaptation could lead to less symptomatic infection and more efficient person-to-person transmission.

## VIROLOGY

Many researchers have indicated that the novel H7N9 virus could cross-species from poultry to humans and increase the pandemic risk as it possesses mammalian adaptation by having mutations in the receptor binding site of the hemagglutinin gene, where Gln226Leu and Gly186Val substitutions in the hemagglutinin gene have caused higher binding affinities toward human α-2,6-linked sialic acid receptors in the human upper respiratory tract (**Table 1**; Connor et al., 1994; Ha et al., 2001; Chutinimitkul et al., 2010; Wang et al., 2010; Gambaryan et al., 2012; Herfst et al., 2012; Xiong et al., 2013b). To et al. (2013) indicated that human Influenza A viruses bind preferentially to α-2,6-linked sialic acid receptors which are abundant in the human upper respiratory tract, while avian influenza A viruses with 226Glu have higher affinities to avian α-2,3-linked sialic acid receptors in the human lower respiratory tract and avian alimentary tract (Shi et al., 2013c). Hence, the novel H7N9 virus changed its receptor binding properties from avian-type to human-type, which could increase air transmission and pandemic potential. Some novel H7N9 strains have Thr160Ala mutation in the hemagglutinin gene that causes the absence of an *N*-glycosylation site at position 158 and increases the affinity for human receptors (**Table 1**; Wang et al., 2010). Some studies showed that the novel H7N9 virus retains its high affinity toward avian α-2,3-linked sialic acid receptors while having a low but significantly higher affinity to α-2,6-linked sialic acids as compared to other avian H7 subtypes (Dortmans et al., 2013; Ramos et al., 2013). As a result, this novel virus is able to cause avian-to-human transmission by circulating in poultry and binding to the human lower respiratory tract that consists of these two different receptors (Shi et al., 2013a). In short, this novel H7N9 virus consists of the viral-attachment characteristics of both avian and human influenza A viruses, which have never been identified in other avian influenza A viruses. However, further investigation is essential as the novel H7N9 strain A/Shanghai/1/2013 from the first patient did not contain the Gln226Leu mutation but nevertheless had the ability to cause human infections.

**Table 1:** Importance of the key genetic mutations in viral proteins of influenza A H7N9 viruses in the year 2013.

Gene	Mutation	Importance of the mutation	Reference
Hemagglutinin (H3 numbering)	Gln226Leu	Increased binding affinity to α-2,6-linked sialic acid receptor	Connor et al. (1994), Ha et al. (2001), Chutinimitkul et al. (2010), Herfst et al. (2012)
	Gly186Val	Increased binding affinity to α-2,6-linked sialic acid receptor	Gambaryan et al. (2012), Xiong et al. (2013b)
	Thr160Ala	Increased binding affinity to α-2,6-linked sialic acid receptor	Wang et al. (2010)
	Multibasic amino acid at HA0 cleavage site	Cleavage by ubiquitous proteases	Senne et al. (1996), Subbarao et al. (2003)
Neuraminidase (viral release from host cell surface)	Arg292Lys	Neuraminidase resistance	Gubareva et al. (1997), McKimm-Breschkin et al. (1998)
	Deletions in stalk region	Increased virulence	Matrosovich et al. (1999)
PB2 (viral replication)	Asp701Asn	Mammalian adaptation	Li et al. (2005), Gao et al. (2009)
	Leu89Val	Enhanced polymerase activity	Hatta et al. (2001), Labadie et al. (2007)
	E627K	Enhanced viral replication and virulence in mice model	Hatta et al. (2001), Labadie et al. (2007)
PB1 (viral replication)	Ile368Val	Enables droplet transmission in ferrets	Herfst et al. (2012)
PB1-F2 (induce cellular apoptosis and inhibit function of type I interferon)	Full-length	Full-length PB1-F2 needed for virulence in mice	Zamarin et al. (2006)
Matrix protein M1 (viral assembly and budding)	Asn30Asp Thr215Ala	Increased virulence in a mice model Increased virulence in a mice model	Fan et al. (2009) Fan et al. (2009)
Matrix protein M2	Ser31Asn	Amantadine resistance	Hay et al. (1985), Pinto et al. (1992)
NS1 (counteracts host antiviral response)	Pro42Ser Deletion of PDZ-binding motif	Increased virulence in mice Signaling of host proteins	Jiao et al. (2008) Jackson et al. (2008)

There was also a mutation in the PB2 gene segment of the novel H7N9 virus which is responsible for host specificity at which avian-characteristic E (Glutamic acid) at position 627 of PB2 is mutated to human-characteristic K (lysine), which would enhance viral replication and polymerase activity (Dortmans et al., 2013; Kageyama et al., 2013; Knepper et al., 2013; Tharakaraman et al., 2013). Studies showed that the novel H7N9 virus with E627K in PB2 gene caused more efficient viral RNA replications at a lower temperature (33°C), which is the human nasal body temperature, as compared to influenza avian viruses with 627E (**Table 1**; Hatta et al., 2001; Labadie et al., 2007; Dortmans et al., 2013; Knepper et al., 2013; Tharakaraman et al., 2013). Indeed researchers found that the E627K mutation is associated with an increased virulence of the H5N1 virus that is highly pathogenic (Hatta et al., 2001; Labadie et al., 2007). Mutations of both E627K and Asp701Asn in PB2 genes also caused mammalian adaptation of the novel H7N9 virus and efficient respiratory-droplet transmission (**Table 1**; Hatta et al., 2001; Li et al., 2005; Labadie et al., 2007; Gao et al., 2009; Neumann et al., 2012). Moreover, Leu89Val mutation was also detected in the PB2 gene of this novel H7N9 virus that enhanced polymerase activity (**Table 1**; Hatta et al., 2001; Labadie et al., 2007). Additionally, Qi et al. (2014) stated that this novel virus also consists of other human-like signatures such as PA-V100A, PA-K356R, and PA-S409N. However, this novel virus mainly consists of more avian-like residues at signature positions (Qi et al., 2014). Researchers reported that genetic variations in the viral ribonucleoprotein (RNP) complex which consists of PB2, PB1, and PA; would improve cross-species transmission to humans. For instance, mutations of R591Q, and N701D in the PB2 gene have been found to alter host cell tropism (Qi et al., 2014). Past research also revealed that this novel H7N9 virus consists of a full-length of PB1-F2 protein, which is 90 amino acids; which then results in increased virulence (**Table 1**; Zamarin et al., 2006).

There were no multibasic amino acids detected but a single amino acid R at the proteolytic cleavage site of its hemagglutinin gene, which caused this novel virus to display low pathogenicity in poultry (**Table 1**; Senne et al., 1996; Subbarao et al., 2003). This multibasic amino acid motif is a key virulence marker for highly pathogenic avian influenza subtypes H5 and H7 (Senne et al., 1996; Subbarao et al., 2003). Therefore, the novel H7N9 virus has the potential to cause an even more disastrous pandemic via subsequent mutations to acquire multibasic amino acid motifs since it is already highly pathogenic among humans without multibasic amino acids. Experimental studies reported that there were five amino acid deletions at positions 69–73 in the stalk region of NA which caused enhancement in poultry adaptation as viral virulence is associated with the stalk length (Matrosovich et al., 1999). Researchers found that a premature stop codon near the C-terminus of NS1 gene segment caused PDZ domain-binding motif deletion, which attenuates novel H7N9 in mammals (**Table 1**; Jackson et al., 2008). Studies also found that some novel H7N9 strains such as A/Shanghai/1/2013, consisted of Arg292Lys-resistant mutation in the NA gene; which reduced susceptibility to NA inhibitors (NIs) such as zanamivir by 30-fold, and oseltamivir by 100-fold based on a fluorescence-based NA inhibition assay (Gubareva et al., 1997; McKimm-Breschkin et al., 1998, 2003; **Table 1**). However, only one H7N9 strain consisted of Arg292Lys based on current research (McKimm-Breschkin, 2013). Hence, NIs are still used as first-line drugs in treatment as genetic analysis, phenotypic assessment, NA inhibition assay, and *in vitro* testing have confirmed the sensitivity of most strains of H7N9 to oseltamivir and zanamivir (Peng et al., 2013; Shi et al., 2013a; WHO, 2013). In some patients treated with NA inhibitors, resistant H7N9 variants have been detected encoding R292K mutation in NA that confers resistance to oseltamivir (Hu et al., 2013; Wu et al., 2013; Yen et al., 2013; Lin et al., 2014; Shen et al., 2014). Usually the oseltamivir-resistant (NA-R292K) mutations cause reduced viral fitness (Govorkova, 2013); but a study by Hai et al. (2013) did not detect the effect of the NA-R292K mutations in an H7N9 virus in regard to its virulence in mice, thus suggesting that oseltamivir-resistant H7N9 viruses could be competitive in nature. Therefore the continuous surveillance of oseltamivir-susceptibility is essential. Further investigations are also needed as it was found that corticosteroid therapy caused the presence of Arg292Lys mutation in patients (Li et al., 2014).

Researchers successfully identified Asn30Asp and Thr215Ala mutations in the M1 protein of all isolated H7N9 strains which resulted in increased viral virulence (Fan et al., 2009). Moreover, the viral M2 gene of the novel H7N9 consisted of a Ser31Asn mutation that conferred resistance toward M2 channel blockers, amantadine and rimantadine (**Table 1**; Hay et al., 1985; Pinto et al., 1992). Furthermore, the six internal genes of the novel H7N9 virus originated from the H9N2 virus that caused this novel virus to have a severe pathogenesis. This was because the H9N2 virus could induce prominent cytokine and chemokine activation in epithelial cells and marcrophages of humans (Shi et al., 2013a). Researchers also discovered that the novel H7N9 virus replicates more efficiently at the human lower respiratory tract which consists of both 2,3-linked and α-2,6-linked sialic acid receptors (Shi et al., 2013a). Furthermore, studies showed that the novel H7N9 virus reproduces efficiently in human alveolar tissue as its NS1 protein suppresses the response of antiviral beta interferon-type I, which then causes severe lower respiratory tract diseases in infected individuals (Knepper et al., 2013). Moreover, Pro42Ser mutation was identified in its NS1, which increased viral virulence (**Table 1**; Jiao et al., 2008).

There is, however, no sustained human-to-human transmission of the novel H7N9 even though it has acquired human adaptation (Richard et al., 2013). Recent studies also indicated that avian H7N9 tends to bind to lower pulmonary epithelial cells where both α-2,3-linked and α-2,6-linked sialic acid receptors are present, instead of the epithelial cells of the upper respiratory tract; which makes it incapable of causing a pandemic, respiratory droplet-based transmission and efficient transmission between humans (Qi et al., 2013; van Riel et al., 2013). Other studies discovered that the novel H7N9 virus binds inefficiently to human tracheal epithelial cells as compared to seasonal or pandemic avian influenza viruses, which blocks human-to-human transmission (Dortmans et al., 2013). Furthermore, researchers indicated that this novel H7N9 virus is incapable of causing human-to-human transmission because it possess a high pH for the fusion of the hemagglutinin gene, which results in lower thermostability and hemagglutinin stability (Richard et al., 2013).

Therefore, this novel H7N9 virus needs to mutate key signature amino acid residues in the protein functional domains in order to achieve avian-to-human transmission. All findings state that the novel H7N9 virus remains lowly pathogenic and undetectable in poultry. This would create an infection reservoir that increases the potential of the novel H7N9 virus to evolve silently and become highly pathogenic in both poultry and humans in the future (Gao et al., 2013b). Based on these findings, comprehensive and enhanced surveillance is essential among poultry and humans to monitor the evolution and mutation of the novel H7N9 virus that might cause a pandemic outbreak due to highly efficient human-to-human transmission with asymptomatic infections in the future.

## CLINICAL CHARACTERISTICS

It was found that more than 70% of infected patients had exposure to poultry 3–8 days before the onset of illness (Shi et al., 2013a). Initial symptoms observed were generally similar to H1N1 and H5N1 virus infections such as fever, influenza-like illness, and symptoms of lower respiratory tract infection such as productive cough, sputum and dyspnoea (Gao et al., 2013a; Peng et al., 2013; Shi et al., 2013a; WHO, 2013). Typical and atypical antibiotics were also not effective at treating progressive pneumonia in infected patients (Shi et al., 2013a; To et al., 2013). This novel virus predominantly affects the human lower respiratory tract (Shi et al., 2013a); and studies have found that patients who start their treatment later than 5 days after the onset of illness tended to have moderate or severe ARDS (Gao et al., 2013a).

Laboratory investigations revealed that patients had lymphopenia, leucopenia, thrombocytopenia, impaired renal or liver function, hypoxaemia, high chemokine concentrations or serum cytokine, high D-dimer concentrations, disseminated intravascular coagulation, and leucocytosis with neutrophilia as the disease progressed (Lu et al., 2013; Shi et al., 2013a; To et al., 2013; WHO, 2013). The white blood cell count in infected patients was found to be normal or slight lower at the initial stage (Gao et al., 2013a; Lu et al., 2013; Shi et al., 2013b); while the concentrations of C-reactive protein, hepatic aminotransferases, creatine kinase isoenzymes, and lactate dehydrogenase or creatine kinase were raised at some stage of the disease (Gao et al., 2013a; Shi et al., 2013a,b). Furthermore, results from CT scans and chest radiographs revealed diffuse alveolar opacities, multi-lobar patchy consolidation, mediastinal emphysema, and pleural effusions with ground glass changes in infected patients (Gao et al., 2013a; Lu et al., 2013; Shi et al., 2013a,b; To et al., 2013; WHO, 2013). The most common complications noted were ARDS, respiratory failure, refractory hypoxemia, encephalopathy, rhabdomyolysis, multiorgan dysfunction syndrome, secondary bacterial infections, and septic shock which could lead to death (Peng et al., 2013; Shi et al., 2013a,b; To et al., 2013; WHO, 2013). Other than that, clinical investigation found that lymphocytopenia and thrombocytopenia were prognostic indicators of ARDS and death, while most of the infected H7N9 patients died because of refractory hypoxemia (Gao et al., 2013a). The level of C-reactive protein is also used as a clinical marker for illness severity of human H7N9 infections (Shi et al., 2013a).

The novel H7N9 virus has an estimated mean incubation period of approximately 3 days, and more than 95% of infected individuals displayed symptoms within a week of infection. Therefore, the medical surveillance and quarantine implemented on close contacts should be reduced to less than 1 week. The fatality risk was about 33% based on confirmed laboratory-cases (Cowling et al., 2013). The virus has a median time from illness onset to admission, from illness onset to laboratory confirmation, from hospital admission to discharge, and from hospital admission to death of about 4.5, 8.3, 41.7, and 11 days respectively (Cowling et al., 2013; WHO, 2013). These statistics, however, should not be applied globally to other countries since these estimations would be different based on the medical advancement in different countries. For instance, countries with more advanced surveillance systems and medical services will be able to detect this novel virus in a shorter time, sustain the life of patients for a longer time, as well as shorten the recovery period.

## TREATMENT, CONTROL, AND PREVENTION

There is currently no effective vaccine available for the management of this virus (Qi et al., 2014). According to several randomized clinical trials involving the pediatric, adult and geriatric populations; the NA inhibitors, namely oseltamivir, zanamivir, and peramivir; have shown good efficacy in the management of the novel H7N9 virus (Hayden et al., 1997, 1998, 1999; Monto et al., 1999; Hedrick et al., 2000; Nicholson et al., 2000; Treanor et al., 2000; Lalezari et al., 2001; Whitley et al., 2001; Sato et al., 2005; Cowling et al., 2010; Heinonen et al., 2010; Yu et al., 2010; Hernán and Lipsitch, 2011). However, the novel H7N9 virus confers resistance to both rimantadine and amantadine which are M2-ion channel blockers, and as such they are not recommended for use in treatment (Li et al., 2014; Qi et al., 2014). Previous studies using hyperimmune IV immunoglobulin for the treatment of severe influenza A (H1N1) infection resulted in a lower viral load and reduced mortality (Zheng et al., 2008). This prompted investigators to propose the role of such a treatment for the management of severe H7N9 illness as well (Shi et al., 2013a).

According to the guidelines released based on recommendations from the World Health Organization (WHO) and Centers for Disease Control and Prevention (CDC), treatment is warranted for all hospitalized patients, and for confirmed and probable outpatient cases. Treatment should ideally be initiated within 48 h of the onset of illness as it has been shown to reduce disease severity as well as mortality (Jain et al., 2009; Siston et al., 2010; Hiba et al., 2011; Kumar, 2011; Rodríguez et al., 2011; Yu et al., 2011; Chemaly et al., 2012; Hsu et al., 2012; Louie et al., 2012; Muthuri et al., 2013). However, treatment should be initiated even if 48 h has lapsed. For uncomplicated illness, the recommended dose is two doses per day of an NA inhibitor medication, for a period of 5 days. For patients with severe or complicated illness, treatment with oral or enterically administered oseltamivir is recommended, however, data is currently lacking as to the duration of treatment. The CDC in the meantime has currently recommended a longer course of treatment, i.e., 10 days, pending further investigations. Real time-PCR or rapid point-of-care tests which could detect influenza A virus infection, are used for starting antiviral treatments earlier before the subtype is determined. Currently, real time-PCR is used to detect and confirm novel H7N9 infections (Shi et al., 2013b).

Some control measures should be taken to prevent further animal-to-person transmission such as the temporary closure of live poultry and bird markets in infected regions. Indeed the closure of live poultry markets was found to reduce human H7N9 infections drastically in affected provinces over the summer in Liu et al. (2013c) and Yu et al. (2013). Besides that, improved biosecurity in animal husbandry, comprehensive surveillance, vaccination programs, poultry culling, disinfection, and the segregation of different species in poultry markets are essential in order to prevent the novel H7N9 virus from evolving and causing a pandemic (Liu et al., 2013a; Shi et al., 2013a; Li et al., 2014). Moreover, live poultry should not be kept overnight and regular rest days for live poultry markets should be implemented (Yu et al., 2013). Some measures such as immediate isolation of infected patients, progressive monitoring of close contacts, and standard safety guidelines for healthcare workers are essential even though there is no sustained human-to-human transmission (Li et al., 2014).

In the long term, a multi-sectoral approach that studies the interrelated relationship between the emergence and transmission of the novel H7N9 virus with the cultural, biological, economic, social, and environmental factors should be highlighted. This is to restructure the system of live poultry market instead of destabilizing them. For example, changes should be made to the practice of poultry trading and purchasing. Food security can then be ensured while the risk of disease transmission can be decreased.

## CONCLUSION

In conclusion, an immediate pandemic outbreak of the novel H7N9 virus among humans is unlikely to happen, based on the above findings, which showed that this novel virus has not acquired all the characteristics of previous human avian influenza viruses that caused efficient human-to-human transmissions. However, these viruses have shown the capability of acquiring mammalian-adapting amino acid changes, thus enabling them to reassort with circulating human viruses (Zhu et al., 2013b). The occurrence of high fitness of oseltamivir-resistant H7N9 viruses also definitely poses a significant threat to humans (Richard et al., 2013; Zhu et al., 2013a). Therefore enhanced surveillance is essential to continue monitoring and characterizing this novel H7N9 virus in order to have a better understanding of the molecular mechanisms which enable the crossing of the species barrier, and identifying the possibility of airborne transmission between humans; to prevent a pandemic outbreak in the future. Medical surveillance and services must also be improved to ensure early detection of human H7N9 infections in order to have a higher rate of recovery. There is currently no vaccine available and NIs are the main antiviral treatments used. Further studies on antiviral treatments and vaccines are essential in order to reduce the associated morbidity and mortality. Moreover, the biosecurity and hygiene of live poultry markets and farms which act as the main source of novel H7N9 infections have to be improved and updated from time to time. This is to stop the human epidemic while preventing the novel H7N9 virus from circulating, spreading, maintaining, and reassorting to evolve as a more pathogenic avian influenza A virus or a pandemic agent.

## Conflict of Interest Statement

The authors declare that the research was conducted in the absence of any commercial or financial relationships that could be construed as a potential conflict of interest.

## Footnotes

1 http://www.cidrap.umn.edu/sites/default/files/public/downloads/topics/cidrap_h7n9_update_051614_0.pdf

2 http://www.info.gov.hk/gia/general/201405/17/P201405170325.htm

3 http://www.chp.gov.hk/files/pdf/2014_avian_influenza_report_vol10_wk19.pdf
